# Acrolein Induces Endoplasmic Reticulum Stress and Causes Airspace Enlargement

**DOI:** 10.1371/journal.pone.0038038

**Published:** 2012-05-31

**Authors:** Yoshiaki Kitaguchi, Laimute Taraseviciene-Stewart, Masayuki Hanaoka, Ramesh Natarajan, Donatas Kraskauskas, Norbert F. Voelkel

**Affiliations:** 1 Pulmonary and Critical Care Medicine Division, The Victoria Johnson Laboratory for Obstructive Lung Disease Research, Virginia Commonwealth University, Richmond, Virginia, United States of America; 2 First Department of Internal Medicine, Sinshu University School of Medicine, Matsumoto, Japan; 3 Division of Pulmonary Sciences and Critical Care Medicine, University of Colorado School of Medicine, Aurora, Colorado, United States of America; University of Giessen Lung Center, Germany

## Abstract

**Background:**

Given the relative abundance and toxic potential of acrolein in inhaled cigarette smoke, it is surprising how little is known about the pulmonary and systemic effects of acrolein. Here we test the hypothesis whether systemic administration of acrolein could cause endoplasmic reticulum (ER) stress, and lung cell apoptosis, leading to the enlargement of the alveolar air spaces in rats.

**Methods:**

Acute and chronic effects of intraperitoneally administered acrolein were tested. Mean alveolar airspace area was measured by using light microscopy and imaging system software. TUNEL staining and immunohistochemistry (IHC) for active caspase 3 and Western blot analysis for active caspase 3, and caspase 12 were performed to detect apoptosis. The ER-stress related gene expression in the lungs was determined by Quantitative real-time PCR analysis. Acrolein-protein adducts in the lung tissue were detected by IHC.

**Results:**

Acute administration of acrolein caused a significant elevation of activated caspase 3, upregulation of VEGF expression and induced ER stress proteins in the lung tissue. The chronic administration of acrolein in rats led to emphysematous lung tissue remodeling. TUNEL staining and IHC for cleaved caspase 3 showed a large number of apoptotic septal cells in the acrolein-treated rat lungs. Chronic acrolein administration cause the endoplasmic reticulum stress response manifested by significant upregulation of ATF4, CHOP and GADd34 expression. In smokers with COPD there was a considerable accumulation of acrolein-protein adducts in the inflammatory, airway and vascular cells.

**Conclusions:**

Systemic administration of acrolein causes endoplasmic reticulum stress response, lung cell apoptosis, and chronic administration leads to the enlargement of the alveolar air spaces and emphysema in rats. The substantial accumulation of acrolein-protein adducts in the lungs of COPD patients suggest a role of acrolein in the pathogenesis of emphysema.

## Introduction

Both active cigarette smoking and chronic exposure to cigarette smoke of non-smokers in enclosed environments, so-called second hand smoke exposure, cause heart and lung diseases in susceptible individuals [1]–[4]. Inhalation of cigarette smoke introduces exogeneous reactive oxidants into the airways and also causes generation of endogenous oxidants released from phagocytes and other cells in the lungs [5], [6]. It has been long appreciated that cigarette smoke contains particles, volatile components and endotoxin [7], [8] and that a multitude of individual smoke components, or interactions between a number of these components, are responsible for the chronic respiratory bronchiolitis [9] and emphysematous destruction [10] of the lung. Stedman reported 40 years ago that cigarette smoke is comprised of more than 4700 chemicals [11] and therefore many investigators consider it a somewhat futile exercise to investigate which of these cigarette smoke components cause inflammation and lung tissue damage. Although the burning cigarette may also be an antigen delivery device [10], there may be chemicals which, when inhaled, have cytotoxic and genotoxic effects. One such compound inhaled with the cigarette smoke is the highly aggressive aldehyde acrolein. Depending on the brand of the cigarette 200–400 µg of this volatile aldehyde are inhaled with the smoke generated by a single cigarette [12]. The ‘dosing’ of aldehyde to the lung is not restricted to the airways via inhalation, because acrolein also appears in the blood of smokers and is excreted in the urine [13]. Systemic effects of acrolein are likely also to occur following uptake via the gastrointestinal tract. Acrolein forms protein – and DNA-adducts [14]–[16] and it has been shown that acrolein affects membrane lipids [17]. Of interest, acrolein, like ceramide [18], [19], is also an endogenous metabolic product produced by activated neutrophils [20], [21]. Acrolein has been shown to induce the release of cytokines from human macrophages, and elevated plasma levels of acrolein can be measured as a byproduct of polyamine metabolism in patients with renal failure [22], [23]. Given the relative abundance of acrolein in inhaled cigarette smoke and its recognized toxic potential as a product of activated inflammatory cells, it is surprising how little we know about the pulmonary effects of systemic acrolein levels. We are aware of only one report by Borchers et al. [24] demonstrating that inhalation of acrolein caused lung inflammation and airspace enlargement in rats. An important distinguishing feature of our present investigations is the systemic administration of acrolein. This approach was taken in order to model the effects of circulating acrolein on the rat lung.

Our data demonstrate that acute administration to acrolein induced ER stress response gene expression and upregulated VEGF protein in the lung tissue. The chronic exposure to acrolein caused apoptosis of alveolar septal cells, downregulation of VEGF protein expression and the development of emphysema. There was a significant accumulation of acrolein-protein adducts in the lungs of COPD patients suggestive of a role of acrolein in emphysema pathogenesis. Our findings are important in the context of the toxic effects of second hand smoke and provide evidence for a new molecular mechanism of acrolein cytotoxicity in the lung tissue.

## Results

### Acute effects of systemic acrolein administration

**Figure 1:**
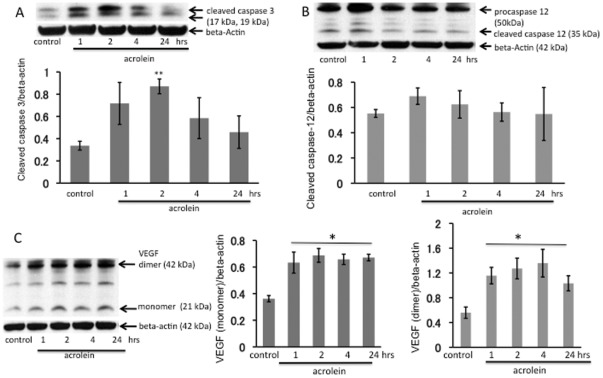
Acute effects of acrolein exposure. Western blot analysis of active cleaved caspase 3 (**A**), caspase 12 (**B**) and VEGF (**C**) in whole lung tissue homogenates. WB bands were normalized to beta-actin. **P<0.01; *P<0.05 when compared to a control group. Rats received a single i.p. injection of acrolein at 12 µmol/kg and were sacrificed 1, 2, 4 or 24 hours after the challenge. n = 4 animals per group, data are mean±S.E.

To assess the acute effects of acrolein on the lung tissue we used a single intraperitoneal injection of acrolein at a dose of 12 µmol/kg in PBS. The rationale for the acute systemic dosing was to begin to explore systemic (circulating) acrolein effects on the lung, because heavy smokers have elevated blood levels of acrolein. Animals were sacrificed at 1, 2, 4 and 24 hour(s) after the acrolein injection. The levels of acrolein injected are compatible with concentration of acrolein in mainstream smoke from one cigarette that is directly exhaled from smoker after taking a puff on a lit cigarette [25]. No inflammatory cell accumulation was observed in any of the lungs. There was a significant increase of cleaved caspase 3 protein in the lung tissue at 2 hours post i.p. injection (Figure 1A) supporting the interpretation that acute systemic administration of acrolein induced transient lung cell apoptosis. In contrast, there was no increase in lung tissue cleaved caspase 12 (Figure 1B). One single injection of acrolein was followed by a persistent increase in lung tissue VEGF protein expression (Figure 1C). Similar effects of acrolein have been documented in cultured endothelial cells treated with tunicamycin, a known inducer of endoplasmic reticulum (ER) stress [26].

**Figure 2:**
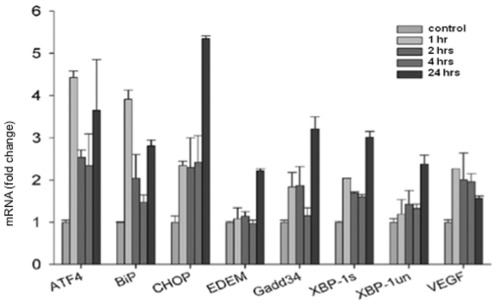
Quantitative real-time PCR analysis of the Unfolded Protein Response (UPR)-related gene expression. Rats received a single i.p. injection of acrolein at 12 µmol/kg and were sacrificed 1, 2, 4 or 24 hours after the challenge. n = 4 animals per group, data are mean±S.E.

In order to investigate the acute effects of acrolein on signals representing ER stress we chose the transcription factor ATF4, BiP, a chaperone, CHOP- a transcription factor down-stream from ATF4, Gadd 34 (growth arrest and DNA damage) which is induced by a variety of stressors [27] and XBP-1 (X-box binding protein-1) which has been associated with ER-stress-triggered phospholipid biosynthesis. As shown in Figure 2 acrolein induced a significant early upregulation of ATF4 and BIP transcripts and later, at the 24 hours after injections resulted in significant induction of CHOP and to a lesser degree of ARF4, BIP, Gadd34 and Spliced form of X-box binding protein-1 (*XBP-1s*) transcription.

### Systemic chronic administration of acrolein causes airspace enlargement

To examine the effects of chronic systemic challenges with acrolein on the integrity of the lung tissue we administered 3 different doses of acrolein once a week for 3 weeks.

**Figure 3:**
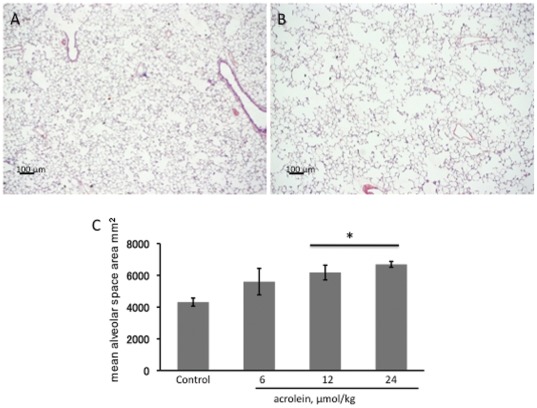
Histology of rat lungs. **A.** Section of a representative control rat lung showing normal alveolar structure. **B.** Section of a lung from a 24 µmol/kg acrolein treated rat showing enlarged airspaces. Magnification: 25×. Scale bars = 100 µm. **C.** Emphysema in acrolein treated lungs assessed by mean alveolar airspace area (µm^2^). Data generated from n = 4 animals/per group, values are mean±S.E. * P<0.05 compared with the control group

When compared to control rat lungs (Figure 3A), the lungs from rats treated with a single injection of acrolein (24 µmol/kg) once a week for 3 consecutive weeks show diffuse airspace enlargement (Figure 3B) without obvious inflammatory responses. As measured by the mean alveolar airspace area, chronic systemic acrolein induced a dose-dependent increase of airspace enlargement, (Figure 3C).

### Chronic acrolein treatment causes apoptosis of lung alveolar septal cells

**Figure 4:**
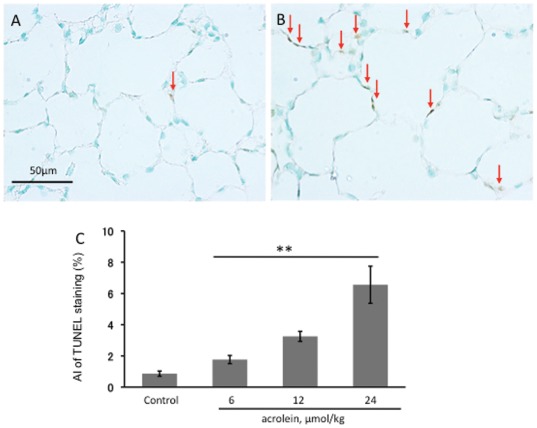
TUNEL staining of rat lung sections. **A.** Control rat lung section, showing only oneTUNEL-positive intra-alveolar cell (arrow). **B.** Lung section from a rat treated with 3 injections of 24 µmol/kg acrolein shows abundant TUNEL^+^ cells in the alveolar septa (arrows). Magnification: 400×. Scale bars = 50 µm. **C.** Apoptotic index (AI) of TUNEL staining was performed as described in Materials and Methods. n = 4 rats/per group, values are mean±S.E. ** P<0.01 compared with control rat group.

TUNEL staining performed to screen for the presence of alveolar septal cells undergoing necrosis or apoptosis revealed, that when compared to control lungs (Figure 4A), there were a large number of labeled TUNEL+ cells in the lungs from acrolein-injected animals (Figure 4B). The quantification of TUNEL data is presented in Figure 4C.

**Figure 5:**
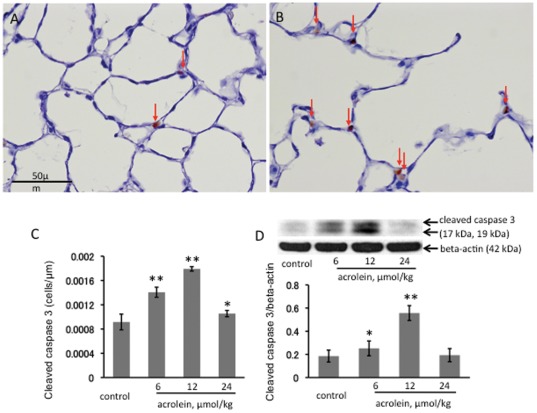
Immunohistochemistry for cleaved caspase 3 counterstained with hematoxylin. **A.** Control rat lung, showing a few caspase 3 positive cells in the alveolar septa (arrows). **B.** Lung tissue section from a set of treated with 12 µmol/kg acrolein, showing caspase 3 positive cells in the alveolar septa (arrows). Magnification: 400×. Scale bars = 50 µm. **C.** The apoptotic Index (AI) of immunohistochemistry for cleaved caspase 3 was calculated as described in Materials and Methods. **D.** Western blot analysis of cleaved caspase 3 protein in the lung tissue extracts. n = 4 rats/per group, values are mean±S.E. *P<0.05 and **P<0.01 when compared to the control group.

Immunohistochemistry for caspase 3 expression showed that, when compared to control lungs (Figure 5A), number of cells positive for caspase 3 expression is much higher in acrolein-treated rat lungs (Figure 5B). The apoptosis index based on caspase^+^ cells per 10 high power microscopic fields was greater in the 12 µmol/kg acrolein-treated group when compared to the 6 µmol/kg-treated group (Figure 5C). Of interest, the injection of the highest dose of 24 µmol/kg of acrolein did not cause a further increase in the number of caspase+ cells-perhaps indicating that at the time the animals had been sacrificed this acrolein dose had caused cell necrosis and fewer cells were undergoing apoptosis. The IHC data were confirmed by Western blot analysis. Cleaved caspase 3 protein expression was increased in the lungs from animals treated with the 12 µmol/kg dose of acrolein, but not the 24 µmol/kg dose (Figure 5D).

### Chronic acrolein treatment: VEGF and ER stress responses in the lung

**Figure 6:**
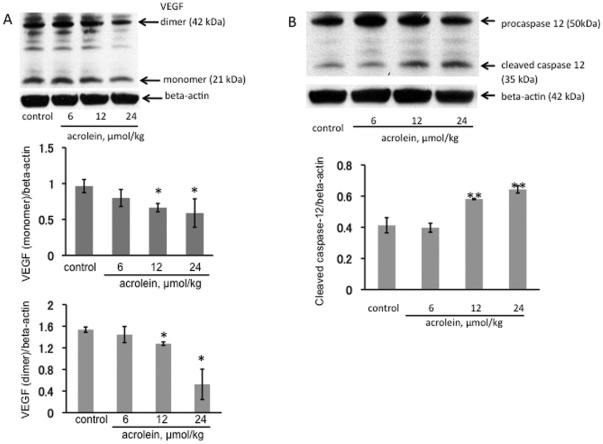
Western blot analysis of VEGF (A) and caspase 12 (B) in lung tissue homogenates from chronically acrolein injected rats. The VEGF (dimer and monomer) protein expression was normalized to beta-actin. *P<0.05; **P<0.01 when compared to the control group.

**Figure 7:**
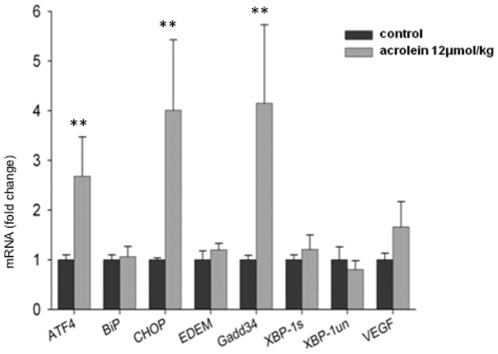
Quantitative real-time PCR analysis of Unfolded Protein Response (UPR)-related gene expression in the 2 experimental rat groups (chronic challenge). n = 4 rats/per group, values are mean±S.E. **P<0.01 when compared to the control group.

The VEGF protein expression in the lungs from acrolein treated animals was assessed by Western blotting. There was a dose-dependent decrease in the amount of lung tissue VEGF protein levels (Figure 6A) that correlates with the loss of the lung structure and the development of emphysema. This is in agreement with previously published data of rat models of emphysema where lung tissue VEGF protein loss has been reported [28]. Treatment with 12 and 24 µmol/kg acrolein injections also significantly increased levels of active cleaved inflammatory caspase 12 (Figure 6B) when compared to control or 6 µmol/kg acrolein treatment. Moreover, chronic acrolein (12 µmol/kg) administration also resulted in a statistically significant increase in the mRNA levels of the ER stress response proteins ATF4, CHOP, and Gadd 34 (Figure 7) in the lung tissue.

### Acrolein-Protein adducts in COPD Patient lungs

**Figure 8:**
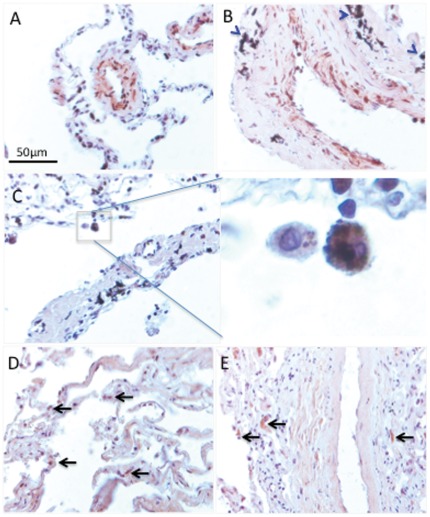
Acrolein-protein adducts in COPD (A–C) and healthy (D, E) lungs. In COPD lungs there is an abundant presence of acroleinated proteins in endothelial and smooth muscle cells of the small vessels (A), bronchoepithelial cells (B), and inflammatory cells (C). The enlarged section (C) shows intense staining of alveolar macrophages. Immunohistochemistry was performed on paraffin-embedded lung sections using a rabbit polyclonal anti-acrolein antibody (Novus Biologicals). There are very few acrolein-protein adducts in healthy lungs (D, E).

We found marked staining in the lungs from patients with COPD when using the antibody directed against acroleinated proteins. As shown in Figure 8A–C, acroleinated proteins were found in bronchoepithelial (A), endothelial and smooth muscle cells of the small vessels (B) and inflammatory cells (C). Blue arrowheads indicate tar deposition in smokers with COPD lungs. The enlarged area from Figure 8C shows macrophages with engulfed apoptotic bodies. The representative image reflects observation made from 6 different patient samples. It is possible that acrolein-protein adducts contribute to the impairment of macrophage function in emphysema. In contrast, in healthy lungs, there we found very few inflammatory cells (arrows) staining positive for acrolein-protein adducts (Figure 8D and 8E).

## Discussion

The highly reactive aldehyde acrolein is a component of inhaled cigarette smoke. Acrolein can be produced endogenously as a metabolic product of activated inflammatory cells [22], [29], and cyclophosphamide-induced lung toxicity is possibly due to acrolein-adduct formation [30]. Our study shows that acute acrolein treatment increases VEGF gene and protein expression. This effect declined with chronic treatment. Our findings are in agreement with the reported elevation of VEGF in the sputum of healthy smokers and with the decrease of both VEGF and VEGF receptor expression in COPD and with the *in vitro* study by Volpi et al demonstrating increased production of VEGF in pulmonary smooth muscle cells and fibroblasts in response to cigarette smoke extract, acrolein, crotonaldehyde and 4-HNE treatments [31]. Upregulation of VEGF production might reflect cells initial defensive response against injury.

However, chronic administration of acrolein causes airspace enlargement due to lung cell apoptosis and loss of lung tissue VEGF expression. Interestingly, the acrolein-dependent lung cell damage was not accompanied by inflammation. In addition, we show that acrolein induces ER stress in the lung. In earlier studies acrolein-induced ER/UPS stress has been documented only in the cell cultures [32], [33]. Although it has been generally accepted that acrolein is toxic, we find only a single publication which demonstrates that inhaled acrolein causes lung inflammation characterized by macrophage and lymphocyte accumulation and airspace enlargement [24]. The findings, presented here, are to our knowledge the first data that show that systemic challenge with acrolein causes activation of lung cells within hours of its injection, and that multiple systemic injections cause a dose-dependent airspace enlargement (Figure 3). Because the highest dose of acrolein injected caused the most abundant increase of TUNEL+ cells, yet was associated with a less abundant labeling of activated caspase 3+ cells, we speculate that this highest dose caused both apoptosis and necrosis since TUNEL+ cells can also be cells which undergo necrosis.

Acrolein induced pulmonary function changes in rats consistent with emphysema have been reported by Costa et al [34]. Acrolein effects on the lung were recently reviewed by Bein et al [29]. Acrolein was found in sputum and exhaled condensate from COPD patients [35]. Acrolein has been shown to induce VEGF [31] and GADD45 beta [36] transcripts as well as increase apoptosis and inhibit VEGF mobilization [37], [38]. There is a report suggesting that glutathione conjugation effectively removes acrolein from external exposures such as cigarette smoke [39].

Apoptosis sensitivity or resistance to acrolein may be cell-type specific; for example 100 µM acrolein caused neuronal cell death after 4 hours [40], and cultured endothelial cells underwent apoptosis when challenged with 10 µM of acrolein [41]. It has been also reported that the antiangiogenic effects of the alkylating chemotherapeutic drug cyclophosphamide are likely due to the release of acrolein [42]. One limitation of our study is that we did not measure tissue acrolein levels. However, we can assume that sufficient amounts of acrolein reached the lung since there was a significant lung tissue remodeling after chronic systemic administration of acrolein.

Kelsen et al. [43] identified endoplasmic reticulum (ER) stress and activation of the unfolded protein response in the lung tissue from patients with COPD. Prompted by this report [43], we asked whether acrolein exposure generates ER stress and triggers the unfolded protein response in the lung tissue of acrolein-challenged animals. To test whether the lung tissue from acrolein-treated animals expressed markers of ER stress, we extracted RNA from lung tissue at different intervals after the acute acrolein treatment and screened for a panel of genes expressed during ER stress. Proteins enter the ER as unfolded polypeptide chains and cells adjust the protein folding capacity of the ER. Such homeostatic control is achieved through the action of a signal transduction pathway that utilizes sensors facing the ER lumen. ER stress results in one of three responses: first, there is a reduction in the protein load entering the ER; second, there is an increase in the ER capacity to handle unfolded proteins and third, if homeostasis cannot be achieved, cell death is triggered, perhaps to protect organisms from cells which accumulate unfolded proteins [44]. In search of signals representing ER stress we chose the transcription factor ATF4, binding immunoglobulin protein BiP- (a chaperone), cytosine-cytosine-adenine-adenine-thymine enhancer-binding protein homologous protein CHOP) (a transcription factor down-stream from ATF4), Gadd 34 (growth arrest and DNA damage) which is induced by a variety of stressors [27] and XBP-1 (X-box binding protein-1) which has been associated with ER-stress-triggered phospholipid biosynthesis.

Unregulated expression of the transcription factor CHOP promotes cell death, and CHOP deletion protects against the death of ER stressed cells. CHOP activates Gadd 34 and Gadd 34 deletion likewise protects against ER stress-induced cell death [27]. Our data show that acrolein induces ER stress, which can be documented as early as 1 hour after acrolein injection (ATF4, BiP and CHOP mRNA). Multiple acrolein injections, which were associated with alveolar cell loss and airspace enlargement, caused increased expression of ATF4 and of the cell death associated markers, CHOP and Gadd 34 (Figure 5B). Study by Geraghty et al. showed induction of the unfolded protein response via ATF4 mediated induction of C/EBP homologous protein CHOP in primary small airway epithelial cells treated with cigarette smoke extract [45]. Similar to our findings with acrolein treatment, acute exposure to cigarette smoke of mice and guinea pigs led to increased expression of CHOP [45].

While early induction of BIP and XBP-1 by acrolein may play a role in triggering lung cell stress, chronic exposure to acrolein did not affect the expression of these genes. Chronic acrolein treatment induced activating transcription factor 4 (ATF4) and C/EBP homologous protein (CHOP) as signals relating to lung cell apoptosis. In the aggregate our data support the concept that systemic application of acrolein, modeling circulating acrolein, causes lung cell death. These data are similar to data obtained after chronic exposure of rats to cigarette smoke; for example, Wu et al. [46] reported an increased expression of lung tissue Bid, cleaved caspase 3 and p53. Kelsen et al. showed that cigarette smoke extract increases the expression of proteins associated with the unfolded protein response in a human airway epithelial cell line and speculated that the smoking-induced activation of the unfolded protein response may serve as a protective mechanism [43]. Although we find that acrolein increases the expression of ATF4, consistent with the increased expression of ATF4 after cigarette smoke exposure as reported by Kelsen et al. [43], the concomitant large increase in CHOP and Gadd 34 mRNA after acrolein exposure prompts us to propose that acrolein administration had brought to the surface the aspect of the unfolded protein response that accompanies the breakdown of the protective defenses of the lung tissue, leading to apoptosis and tissue destruction.

Although we were surprised to find that the acutely acrolein-induced lung cell death was not associated with a loss of lung tissue VEGF expression (Figure 1B), this fact is not unprecedented and supports our observation that acrolein induces ER stress; Pan et al. showed in hepatoma cells that ER stress (likely via ATF4) increases VEGF transcription [47], Roybal et al. showed increased VEGF transcription in ER stressed retinal pigmented epithelial cells [48] and Li et al. in tunicamycin-stressed retinal capillary endothelial cells [49]. Whether VEGF – in the lungs from acrolein-treated animals- is functional and signals effectively, among other actions, via increased endothelial cell NO and prostacyclin production remains to be investigated. Such concern is justified, because Nana Sinkam et al. demonstrated that acrolein inhibits the expression of the PGI_2_-synthase gene in human pulmonary microvascular endothelial cells [50].

Our data also illustrate that, cigarette-smoke can trigger long-lasting memory responses that persist beyond the immediate period of exposure to cigarette smoke, in the form of acrolein-protein adducts. While there are few acrolein-protein adducts the healthy lungs (Figure 6D, 6E), acroleinated proteins are abundantly labeled in a variety of cells from the lungs of COPD patients (Figure 6A–C). These findings suggest that acrolein-protein adduct formation might impair macrophage function and contribute to the pathogenesis of emphysema.

There are many unsaturated aldehydes in cigarette smoke. Along with acrolein, crotonaldehyde [51] is also accountable for the stimulating effects on cytokine release from pulmonary cells. Acrolein and crotonaldehyde effects are also mimicked by an endogenous highly reactive compound 4-hydroxy-nonenal. This latter substance is an unselective cation channel TRPA1 receptor activator and also an abundant lipid peroxidation end-product found in the lung of COPD patients [52]. Interestingly, 4-hydroxy-nonenal lung adduct level correlated with severity of COPD [52]. Further studies are needed to demonstrate whether other unsaturated aldehydes may cooperate with acrolein in producing human lung emphysema.

## Materials and Methods

### Animals (Acute and chronic systemic acrolein challenge)

All animal use procedures were in strict accordance with the National Institute of Health Guidelines for the Care and Use of Laboratory Animals (IACUC) and approved by the Virginia Commonwealth University's Institutional Animal Care and Use Committee. Male Sprague-Dawley rats, 6 weeks of age were purchased from Harlan (Indianapolis, IN). The animals n = 4 per group were treated with intraperitoneal injections (i.p.) of PBS (control group) or acrolein (in PBS, treatment groups). Acrolein was purchased from Sigma-Aldrich Co. (St Louis, Missouri, USA).

#### Acute acrolein challenge

Twenty four rats were divided into 6 groups. The control group was injected with 900 µl of PBS 1 hour prior to sacrifice. All other animals received a single i.p. injection of 12 µmol/kg acrolein solution and were sacrificed 1, 2, 4 or 24 hours after the acrolein challenge.

#### Chronic acrolein challenge

Rats were divided into 4 groups: Group 1: control group (PBS injection), Group 2: 6 µmol/kg acrolein, Group 3: 12 µmol/kg acrolein, and Group 4: 24 µmol/kg acrolein. The injections were administered intraperitoneally on day 1, 8 and 15. Animals were sacrificed on day 21 after the first injection.

### Tissue processing

After completion of the treatment period the rats were anesthetized with a lethal dose of pentobarbital (Nembutal®). The chest was opened and the cardiopulmonary block was quickly isolated and excised. The right main bronchus was cross-clamped and the left lung was filled with 0.5% low melting agarose in 10% formalin at a constant pressure of 25 cm H_2_O, allowing for homogenous and full expansion of the lung parenchyma [53]. The lungs were then fixed in 10% formalin for 48 hours and paraffin-embedded. Tissue sections from upper and lower lobes of the left lung were used for histological analysis.

### Morphological assessment

The 5-µm paraffin-embedded lung tissue sections were stained with hematoxylin and eosin (HE). 10 random lung fields per tissue section were captured at a 25× magnification using a AxioCam® color camera (Carl Zeiss MicroImaging Inc., Thornwood, New York, USA). AxioVision® Imaging System software (Carl Zeiss MicroImaging Inc.) was used to measure the alveolar airspace areas in pixels per µm^2^. The mean alveolar airspace area of the entire histological section of the lung was calculated using a Microsoft Excel data spread sheet.

### TUNEL staining

Terminal deoxynucleotidyl transferase-mediated dUTP nick end-labeling (TUNEL) was performed using the TACS 2 TdT DAB kit (Trevigen, Gaithersburg, Maryland, USA), following the manufacturer's instructions. Briefly, after deparaffinization and rehydration, sections were digested with proteinase K at a concentration of 20 µg/ml for 15 minutes. Endogenous peroxidase activity was quenched with 3% H_2_O_2_ for 5 minutes. The slides were immersed in terminal deoxynucleotidyl transferase (TdT) labeling buffer (Trevigen). TdT, 1 mM Mn^2+^, and biotinylated dNTP in TdT buffer were then added to cover the sections and incubated in a humid atmosphere at 37°C for 60 minutes. The slides were washed with PBS and incubated with streptavidin-horseradish peroxidase for 10 minutes. After rinsing with PBS, the slides were immersed in diaminobenzidine (DAB) solution (Trevigen). The slides were counterstained for 1 minute with 1% methyl green. To assess the apoptosis index of TUNEL stained tissues, 10 random lung fields per tissue section were captured at a 400× magnification using the AxioCam® color camera (Carl Zeiss MicroImaging Inc.) and the number of TUNEL-positive cells was counted using the AxioVision® Imaging System software (Carl Zeiss MicroImaging Inc.). We focused on TUNEL-positive alveolar septal cells and neglected alveolar macrophages. The number of the TUNEL-positive alveolar septal cells was normalized to the total number of cells in the lung tissue section.

### Immunohistochemistry for caspase 3

Immunolocalization of apoptotic cells was performed on paraffin-embedded, formalin-fixed rat lung sections using a rabbit polyclonal antibody to cleaved caspase 3 (Cell signaling Technology Inc., Beverly, Massachussets, USA). Briefly, after paraffin removal in xylene, the sections were rehydrated and were placed on a plastic container filled with Target Retrieval Solution Citrate pH 6 (Dako, Glostrup, Denmark) and heated in a pressure cooker for 10 minutes after it reached the highest pressure at 125°C. After quenching of endogenous peroxidase with 3% of H_2_O_2_ for 15 minutes, the sections were incubated with rabbit polyclonal antibody to cleaved caspase 3 (1∶200 dilution) for 60 minutes at room temperature. Immunodetection was performed using biotinylated anti-rabbit IgG and peroxidase-conjugated streptavidin (Vector Laboratories, Burlingame, California, USA), with diaminobenzidine (DAB) as the substrate. The slides were counterstained for 30 seconds with hematoxylin.

Caspase-3 positive cells were counted in 10 randomly picked lung fields per tissue section at a 400× magnification using the AxioCam® color camera (Carl Zeiss MicroImaging Inc.) and Apoptotic Index (AI) was calculated as number of caspase-3 positive cells over the total length of alveolar perimeter measured by the AxioVision® Imaging System software (Carl Zeiss MicroImaging Inc.).

### Lung tissue homogenates

The right lung was homogenized immediately after isolation in a buffer containing 150 mM of NaCl, 20 mM of Tris-HCl, 10% of Glycerol, 1% of Igepal CA-630, 1 µg/ml of EDTA, 1 mM of PMSF, 10 µg/ml of Aporotinin, 10 µg/ml of phosphatase inhibitor cocktail (contains L-isozymes of alkaline phosphatase and threonine protein phosphatases (Sigma-Aldrich, Saint Louis, Missouri, USA)), and 1 tablet of protein inhibitor cocktail (contains inhibitors with a broad specificity for aminopeptidases, serine, cystine, an acid proteases (Sigma-Aldrich)). Lung tissue homogenates were centrifuged at 10000 g for 15 minutes, and then supernatants were stored at −20°C until they were used for Western blot analysis.

### Western blot analysis

Proteins (30 µg) were subjected to electrophoresis on 4–12% gradient Bio-Tris gels (Novex, San Diego, California, USA) and transferred to PolyScreen PVDF Transfer Membrane (NEN Life Science Products Inc., Boston, Massachusetts, USA) in Tris-glycine buffer containing 10% methanol. Prestained molecular mass marker proteins (Bio-Rad Laboratories Inc.) were used as standards for the SDS-PAGE. Western blots were visualized using Renaissance Western Blot Chemiluminescence Reagent (NEN Life Science Products Inc.). Antibodies used: caspase 3 (Cell signaling Inc.), VEGF (Santa Cruz Biotechnology Inc., Santa Cruz, California, USA), caspase 12 (Santa Cruz Biotechnology Inc.), β -Actin, anti-rabbit, anti-rat and anti-mouse HRP-conjugated antibodies (Santa Cruz Biotechnology Inc.).

### Real-time quantitative PCR (QPCR) for ER stress markers

Total RNA was extracted from rat lungs and purified using TRI Reagent-LS (Molecular Research Center, Inc., Cincinnati, Ohio, USA) according to the manufacturer's specifications. 1 µg of total RNA was reverse transcribed using the Thermoscript RT-PCR system. Complementary DNA (cDNA) was diluted (1∶500) and real-time QPCR performed using Power SYBR Green QPCR Master Mix (Applied Biosistems, Foster City, California, USA) along with ER stress-related genes (the transcription factor ATF4, binding immunoglobulin protein (a chaperone) BiP, cytosine-cytosine-adenine-adenine-thymine enhancer-binding protein homologous protein CHOP; ER degradation-enhancing alpha-mannosidase-like protein EDEM; growth arrest and DNA damage gene Gadd 34, and X-box binding protein-1 spliced (XBP-1s) and unspliced (XBP-1un) forms; (ATF4, BiP, CHOP, EDEM, Gadd 34, XBP-1s and XBP-1un), as well as VEGF and β-actin primers. Cycling parameters were as follows: 95°C, 10 minutes and 40 cycles of 95°C for 15 seconds and 60°C for 1 minute. A dissociation profile was generated after each run to verify specificity of amplification. All PCR assays were performed in triplicate. No template controls (NTC) and no reverse transcriptase controls (No RT) were included. β-Actin was used as a housekeeping gene against which all the samples were normalized for differences in the quantity of total RNA added to each cDNA reaction and for variation in the reverse transcriptase efficiency among the different cDNA reactions. Automated gene expression analysis was performed using the comparative quantitation module of MxPro QPCR Software (Stratagene, La Jolla, California, USA). The levels of a target gene in test samples were expressed relative to a sample of reference.

### Human subjects and immunohistochemical detection of acrolein-protein adducts

Human tissue was obtained from the University of Colorado Hospital Department of Pathology. Informed consent to use the tissue for research purposes had previously been obtained. Unused transplant donor lungs without evidence of pulmonary disease served as controls. Lungs from three patients with GOLD stage 3–4 and five control lungs were studied. Acrolein-protein adducts in the paraffin embedded lung tissue sections were detected by immunohistochemistry using 2 different antibodies: polyclonal antibody from Novus Biologicals (Littleton, CO) and monoclonal antibody from Abcam (Cambridge, MA) according the manufactures protocols. Both antibodies showed very similar results.

### Statistical analysis

The data were expressed as mean ± SEM. Statistical analysis was performed with the Statview software package (SAS Institute Inc., Cary, N Carolina, USA). The data were normally distributed, and values obtained in the different groups of rats were compared using one-way ANOVA. Statistical difference was accepted at P<0.05.
